# Case Report: Successful management of severe suicidal lamotrigine overdose-induced status epilepticus with sustained low-efficiency dialysis

**DOI:** 10.3389/ftox.2025.1622752

**Published:** 2025-07-31

**Authors:** Naoki Kawahara, Hiroki Matsui, Koji Morishita

**Affiliations:** Trauma and Acute Critical Care Centre, Institute of Science Tokyo Hospital, Tokyo, Japan

**Keywords:** lamotrigine, overdose, blood purification therapy, slow low-efficiency dialysis, case report

## Abstract

**Introduction:**

Lamotrigine, a common antiepileptic, typically has a favorable safety profile. However, an overdose can lead to severe central nervous system complications, including refractory status epilepticus. The optimal management of severe overdose with refractory status epilepticus remains uncertain, and the role of extracorporeal removal methods, such as blood purification, has not been fully established.

**Case Description:**

A 20-year-old female with bipolar disorder presented with altered consciousness and status epilepticus 7 h after ingestion of 4.9 g of lamotrigine. As a case of lamotrigine intoxication, initial management with gastric lavage, activated charcoal, benzodiazepines, and levetiracetam failed to control the seizures, and the patient required continuous midazolam and ventilation. Slow low-efficiency dialysis (SLED) was initiated 18 h after admission. Lamotrigine levels substantially decreased from 33.9 to 13.5 μg/mL within 5 h post-SLED, representing a 60.2% reduction. The patient showed marked neurological improvement, with seizure cessation, allowing for discontinuation of midazolam. She was extubated on day 4 and discharged on day 7 with full recovery.

**Discussion:**

This case highlights the successful use of SLED in severe lamotrigine overdose-induced refractory status epilepticus. The correlation among SLED use, reduced lamotrigine levels, and clinical improvement suggests that blood purification may be beneficial in severe toxicity, especially in patients with status epilepticus. This adds to the evidence supporting blood purification for accelerated drug removal and improved outcomes in select severe cases. Prompt recognition of this potentially life-threatening condition and consideration of intensive care, including blood purification, are vital for successful management.

## 1 Introduction

Lamotrigine is an antiepileptic drug, commonly prescribed for managing seizures and bipolar disorders, known for its favorable tolerability and low incidence of adverse effects (Li et al., 2024; Agrawal et al., 2019). Lamotrigine overdose can induce a spectrum of symptoms, ranging from sensory disturbances and altered consciousness to cardiac effects such as arrhythmias (Mae et al., 2025). Although approximately half of overdose cases have been reported to result in no or only mild symptoms, severe complications—though rare—may include critical central nervous system depression, life-threatening arrhythmias, and cardiac arrest (Mae et al., 2025; Alyahya et al., 2018; Nogar et al., 2011; Lofton and Klein-Schwartz, 2004). The optimal management of severe lamotrigine overdose, particularly in patients presenting with refractory status epilepticus, remains undetermined (Li et al., 2024). Although supportive care and gastrointestinal decontamination are standard initial treatments, sodium bicarbonate and lipid emulsion have also been used in the management of wide-QRS complex dysrhythmia, with varying degrees of success (Alyahya et al., 2018). In contrast, the efficacy of extracorporeal removal methods, such as blood purification therapy, in severe lamotrigine poisoning has not been fully established (Agrawal et al., 2019). Case reports suggest that blood purification therapy can reduce lamotrigine levels (Li et al., 2024; Agrawal et al., 2019). However, its effectiveness in preventing severe outcomes, such as status epilepticus, remains uncertain, and the optimal modality of extracorporeal removal therapy has yet to be determined. The optimal timing for initiating therapy and the potential for rebound phenomena are key aspects that require further elucidation. Refining blood purification protocols based on these factors is crucial for improving outcomes in severe lamotrigine poisoning.

Here, we present the case of a 20-year-old female with severe lamotrigine overdose-induced status epilepticus that was successfully managed with blood purification therapy using slow low-efficiency dialysis (SLED). Ethics approval was not required by our institution, and written informed consent for publication of this case was obtained from the patient.

## 2 Case description

A 20-year-old female with a history of bipolar disorder managed with lamotrigine presented to our emergency department with altered consciousness following a lamotrigine overdose. Earlier that day, after a family disagreement in the morning, she experienced emotional distress and suicidal ideation, which led to an intentional overdose of lamotrigine. She messaged her mother to disclose the overdose around noon. When her mother responded shortly after, the patient was vomiting and unresponsive, with empty packages of lamotrigine 100 mg (49 tablets) and rebamipide 100 mg (2 tablets) nearby. Seven hours after the overdose, the patient remained unresponsive, prompting her mother to call for emergency services and she was brought to our hospital.

Upon examination, the patient’s vital signs were as follows: Glasgow Coma Scale score E4V1M1, blood pressure, 120/90 mmHg; heart rate, 120 bpm; respiratory rate, 24 breaths per minute, and SpO_2_ 96% on room air. Neurological examination revealed hyperreflexia in the patellar tendon reflexes and ankle clonus accompanied by decreased muscle tone in all extremities. The pupils were reactive, and no nystagmus was observed. The remaining physical examination, including respiratory and cardiovascular auscultation, were unremarkable.

Initial laboratory findings included a venous blood gas with a pH of 7.321, pCO2 of 42.5 mmHg, HCO3- of 21.3 mmol/L, and lactate of 5.8 mmol/L, indicating metabolic acidosis with elevated lactate. A complete blood count revealed a white blood cell count of 16,700/mL with 93.9% neutrophils. The biochemistry results were remarkable, with a slightly elevated creatine kinase level of 256 U/L. The serum creatinine level was 0.56 mg/dL, and the blood urea nitrogen level was 11.9 mg/dL, indicating normal renal function. Serum electrolyte levels, liver function tests, and coagulation profiles were within normal limits except for mild hypernatremia (147 mEq/L) and hypokalemia (3.3 mEq/L). Serum lamotrigine levels could not be measured in the hospital and were therefore unknown at that time. Electrocardiography showed sinus tachycardia with a heart rate of 114 bpm, a prolonged QRS duration of 0.118 s, and a corrected QT interval of 0.456 s. Computed tomography of the head revealed no acute intracranial abnormalities.

Based on her history of intentional lamotrigine ingestion and the clinical presentation of altered consciousness and seizures, the patient was diagnosed with lamotrigine toxicity.

**FIGURE 1:**
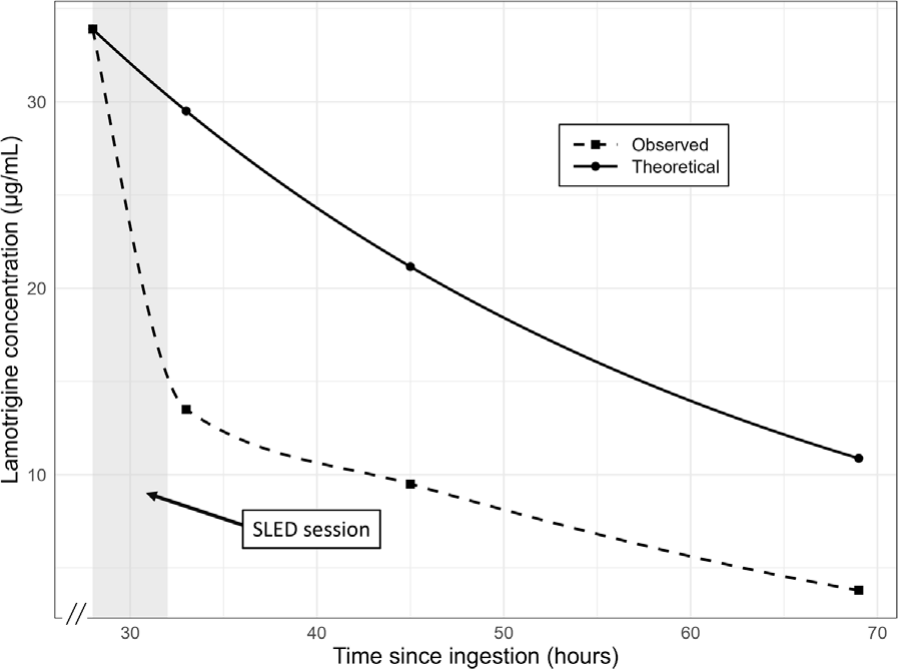
Plasma concentration–time profile of lamotrigine following ingestion. The dashed line represents the theoretical mono-exponential elimination model based on the known half-life of lamotrigine. The solid line corresponds to the observed concentration–time course interpolated using a piecewise cubic Hermite polynomial to ensure passage through each measured point. The abscissa denotes time since ingestion (hours) and the ordinate denotes lamotrigine concentration (µg/mL). SLED: slow low-efficiency dialysis.

Upon arrival, initial management included gastric lavage with an orogastric tube and administration of activated charcoal. To manage subsequent generalized tonic-clonic seizures, intravenous diazepam 5 mg was administered twice, effectively terminating the seizures. Intravenous levetiracetam (500 mg) was administered as an antiepileptic drug. Following drug administration, she was admitted to the hospital; however, her level of consciousness reduced, and she continued to experience intermittent seizures. Continuous intravenous midazolam infusion was initiated at 1–2 mg/h, which reduced seizure frequency but did not eliminate them. Twelve hours after admission, the patient was intubated for airway protection and placed on mechanical ventilation. After intubation, the midazolam infusion was increased to 6 mg/h, resulting in the complete cessation of seizures. Given the severity of her condition, blood purification therapy was selected as the next treatment option after consultation with clinical pharmacists. The primary therapeutic goal was the efficient removal of lamotrigine, a small-molecule drug. Our choice of modality was guided by two main factors. First, to maximize drug removal, a diffusion-based therapy was preferred over convection-based methods like continuous veno-venous hemofiltration (CVVH), as diffusion provides superior clearance of small molecules. Second, the selection between diffusion-based therapies was influenced by our hospital’s manpower limitations, which constrained the use of conventional intermittent hemodialysis. Therefore, SLED was selected. It was initiated approximately 18 h after admission and performed as a single 4-h session. No adverse events were observed during SLED. Serial lamotrigine serum levels were measured (Figure 1; Table 1): the first level before SLED initiation, measured 18 h after admission (28 h after ingestion), was 33.9 μg/mL. This decreased to 13.5 μg/mL (a 60.2% reduction) 5 h after SLED initiation. Levels continued to fall, reaching 9.5 μg/mL (a 72.0% reduction) at 17 h and 3.8 μg/mL (an 88.8% reduction) at 41 h post-SLED initiation. Following SLED, no further seizures were observed and continuous midazolam infusion was discontinued on hospital day 2. The patient’s neurological status gradually improved after SLED, with an improvement in consciousness to a Glasgow Coma Scale score of E4VTM6. Given the clinical improvement, no additional SLED sessions were performed after consultation with clinical pharmacists. The patient was extubated on hospital day 4, and a subsequent electroencephalogram on hospital day 5 revealed no epileptiform discharges. With no recurrence of seizures and full recovery of consciousness, the patient was discharged on hospital day 7 with recommendations for continued psychiatric follow-up. The patient has remained symptom-free over the subsequent 6 months, with no reported recurrence to date.

**TABLE 1:** Time course of lamotrigine serum levels.

Time since ingestion (hours)	28	33	45	69
Time since admission (hours)	18	23	35	59
Time since SLED initiation (hours)	0	5	17	41
Lamotrigine serum level (μg/mL)	33.9	13.5	9.5	3.8

## 3 Discussion

In this case, after controlling for recurrent seizures with continuous midazolam infusion following intubation, we rapidly reduced the serum lamotrigine concentrations using SLED, achieving prompt clinical improvement. This highlights the potential benefits of blood purification therapies, including SLED, for severe lamotrigine poisoning.

Oral lamotrigine is rapidly absorbed, reaching peak plasma concentrations within approximately 2.2–3 h (Wootton et al., 1997). Lamotrigine undergoes hepatic metabolism chiefly via glucuronide conjugation to form inactive metabolites, which are excreted in urine as 2-N-glucuronide conjugates (Pellock, 1994). Its elimination half-life ranges from 25.4 to 32.8 h (Wootton et al., 1997). Another report described a half-life of 25 h in patients with normal renal function and 50 h in uremic patients (Fillastre et al., 1993). The molecular weight of lamotrigine is 256.09 g/mol, and its plasma protein binding rate is approximately 55%–68% (National Center for Biotechnology Information, 2025; Methaneethorn and Leelakanok, 2020). It has been previously reported that hemodialysis reduces the elimination half-life of lamotrigine (Fillastre et al., 1993). Case reports have described rapid reductions in lamotrigine concentrations following dialysis in patients with lamotrigine poisoning (Lu et al., 2012) and faster neurological recovery following hemodialysis (Agrawal et al., 2019). Li et al. (2024) reported a case of successful treatment of a massive lamotrigine overdose using continuous hemodiafiltration. In this case, the initiation of SLED coincided with a significant drop in lamotrigine levels from 33.9 μg/mL to 13.5 μg/mL within 5 h. This biochemical improvement was accompanied by marked clinical improvement. Figure 1 shows the trend of lamotrigine serum concentrations in this case, along with the predicted concentrations, assuming a half-life of 25 h. This observation aligns with previous reports highlighting the utility of hemodialysis in hastening lamotrigine elimination and improving clinical outcomes.

The successful management of status epilepticus in this case report underscores the critical role of aggressive supportive care in severe lamotrigine overdoses. Although lamotrigine overdose is often benign (Lofton and Klein-Schwartz, 2004), severe ingestion can result in life-threatening central nervous system toxicity, resulting in refractory status epilepticus, as observed in this case. Initial seizure control with benzodiazepines and conventional doses of levetiracetam proved inadequate, necessitating escalating doses of midazolam and, ultimately, mechanical ventilation for airway protection. This approach followed the established guidelines for managing status epilepticus from various causes, emphasizing the rapid escalation of anticonvulsant therapy and securing the airway in cases unresponsive to initial treatment (Meierkord et al., 2010). A case series of lamotrigine overdose reported seizure occurrence rates ranging from 10% to 20% in patients with altered mental status (Hatanaka et al., 2022), highlighting that seizures are significant life-threatening complications of lamotrigine poisoning that can lead to poor neurological outcomes and warrant prompt and aggressive management. This case reinforces the importance of preparedness for intensive care interventions, including high-dose sedative infusions and mechanical ventilation, in the management of severe lamotrigine overdose in patients presenting with status epilepticus.

The successful management of this case also highlights the critical role of clinical pharmacists within the multidisciplinary team. In this instance, they were instrumental in decisions to both initiate and discontinue blood purification therapy. As experts in pharmacokinetics, clinical pharmacists are well-positioned to guide such complex therapeutic decisions. Pharmacist-led medication reconciliation is a recognized strategy to reduce medication errors and ensure patient safety during transitions of care (Stuhec, 2025). This collaborative intervention helps identify and resolve medication-related problems that might otherwise be overlooked (Stuhec and Batinic, 2023). The findings from this case, therefore, support the integral role of clinical pharmacists in managing severe drug overdoses to optimize patient outcomes.

This case report has several limitations. First, blood purification was initiated relatively late, at 18 h post-admission. Although the patient achieved a full recovery, it is possible that earlier intervention could have resulted in a more rapid clinical improvement and a shorter hospital stay. Second, this is a single case report and therefore cannot provide a comparative analysis of SLED versus other extracorporeal therapies like IHD or CVVH. The choice of SLED was primarily guided by hospital-specific manpower constraints and does not imply its superiority over other modalities for managing severe lamotrigine overdose.

The primary clinical implication of this case is that it adds to the growing body of literature supporting blood purification, particularly SLED, as a treatment option for severe lamotrigine poisoning, especially in cases complicated by status epilepticus. Although further studies are needed to define optimal protocols and patient selection criteria, this case report suggests that blood purification can be effective in hastening drug removal and facilitating neurological recovery in carefully selected patients.

## 4 Conclusion

This case highlights the successful management of a 20-year-old female who presented with refractory seizures and altered consciousness following a significant lamotrigine ingestion. After the initiation of SLED, the patient demonstrated marked clinical and biochemical improvements. Serial serum lamotrigine levels significantly decreased, correlating with neurological recovery and the ability to cease continuous midazolam infusion. This case demonstrates that blood purification therapy, particularly SLED, is a valuable therapeutic modality for severe lamotrigine poisoning complicated by status epilepticus. Based on the findings in this case, we recommend early consideration of blood purification therapy for patients presenting with life-threatening complications, such as refractory status epilepticus, that are unresponsive to standard medical treatment. Early intervention to accelerate drug removal appears critical to improving neurological outcomes in such critical cases.

## Data Availability

The original contributions presented in the study are included in the article/supplementary material, further inquiries can be directed to the corresponding author.
